# Reprogramming the Human Gut Microbiome Reduces Dietary Energy Harvest

**DOI:** 10.21203/rs.3.rs-2382790/v1

**Published:** 2023-01-25

**Authors:** Karen D. Corbin, Elvis A. Carnero, Blake Dirks, Daria Igudesman, Fanchao Yi, Andrew Marcus, Taylor L. Davis, Richard E. Pratley, Bruce E. Rittmann, Rosa Krajmalnik-Brown, Steven R. Smith

**Affiliations:** 1 AdventHealth Translational Research Institute, Orlando, Florida; 2 Biodesign Center for Health through Microbiomes, Tempe, AZ; 3 Biodesign Swette Center for Environmental Biotechnology, Arizona State University, Tempe, AZ; 4 School of Sustainable Engineering and the Built Environment, Arizona State University, Tempe, AZ

## Abstract

The gut microbiome is emerging as a key modulator of host energy balance^1^. We conducted a quantitative bioenergetics study aimed at understanding microbial and host factors contributing to energy balance. We used a Microbiome Enhancer Diet (MBD) to reprogram the gut microbiome by delivering more dietary substrates to the colon and randomized healthy participants into a within-subject crossover study with a Western Diet (WD) as a comparator. In a metabolic ward where the environment was strictly controlled, we measured energy intake, energy expenditure, and energy output (fecal, urinary, and methane)^2^. The primary endpoint was the within-participant difference in host metabolizable energy between experimental conditions. The MBD led to an additional 116 ± 56 kcals lost in feces daily and thus, lower metabolizable energy for the host by channeling more energy to the colon and microbes. The MBD drove significant shifts in microbial biomass, community structure, and fermentation, with parallel alterations to the host enteroendocrine system and without altering appetite or energy expenditure. Host metabolizable energy on the MBD had quantitatively significant interindividual variability, which was associated with differences in the composition of the gut microbiota experimentally and colonic transit time and short-chain fatty acid absorption in silico. Our results provide key insights into how a diet designed to optimize the gut microbiome lowers host metabolizable energy in healthy humans.

In recent years, it has become irrefutable that the microbial communities in the colon have a profound effect on host physiology, including immune function, inter-organ communication, and metabolism^3^. The majority of studies in humans have correlated the gut microbiota’s composition, gene expression, and metabolism with human-health endpoints such as body weight, glycemic control, and inflammatory bowel diseases^4,5^. What remains to be determined is whether the gut microbiome is a causal driver of host physiology or merely an association^6^.

The effect of the gut microbiome on weight regulation has been a topic of high interest^1^. Obesity is a major public health problem that is at the nexus of metabolic diseases such as cardiovascular disease, non-alcoholic fatty liver disease, and type 2 diabetes^7^. The gut microbiome has emerged as a control center for host energy balance through its impacts on energy harvest from food, gut hormones, and signaling through metabolites such as short chain fatty acids (SCFAs)^1^. Existing data are largely restricted to preclinical models or observational studies^8–10^. Prior controlled feeding studies have demonstrated that high-fiber diets are associated with reduced host metabolizable energy^11^ and that variable energy load can alter energy harvest efficiency in a way that correlates to phyla in the gut microbiota^12^. Despite these advances, a fundamental gap in knowledge is whether or not the gut microbial ecosystem is on the causal pathway leading to weight regulation in humans. Studies to date also lack a comprehensive quantitative evaluation of the contribution of the gut microbiome to the entire energy balance equation, including energy intake, energy expenditure, and fecal energy losses. Prior studies were also not sufficiently precise to detect potentially modest differences that can vary dramatically between individuals, particularly when appropriate environmental controls were not implemented.

To address these critical knowledge gaps, we quantified the intersection of host and gut microbiome factors on human energy balance by performing a controlled feeding study in a metabolic ward using a deep-phenotyping paradigm of quantitative bioenergetics (NCT02939703)^2^ (Extended Data Fig. 1a–b). The intervention included a highly digestible control Western Diet (WD) and a Microbiome Enhancer Diet (MBD). The MBD was designed to maximize the availability of dietary substrates to the gut microbiome and included these four drivers: dietary fiber, resistant starch, large food particle size, and limited quantities of processed foods (Extended Figure 1a). Our design provided equivalent metabolizable energy and total macronutrients (fat, protein, carbohydrates) based on classic principles and equations of food digestibility^13^. Diets were prepared in our metabolic kitchen and validated by measuring energy content via chemical analysis. The details of participant flow from enrollment through analysis are detailed in Extended Data Fig. 1c.

To avoid the confounding effects of energy imbalance on host and microbial metabolism, the diet intervention maintained each participant in energy balance. Energy balance, evaluated by real-time energy intake and energy expenditure (measured via whole-room indirect calorimetry), was maintained within our target of +/− 50 kcals per 6-day calorimeter stay (WD 4.1 ± 5.1 kcal/day; MBD 5.4 ± 2.8 kcal/day; p = 0.8) (Extended Data Fig. 2a). Weight stability was a secondary criterion for evaluating energy balance, and we previously reported that weight was stable during the 6-day calorimetry assessment period whilst the primary endpoint was measured; the study team members were blinded to the diet assignment^2^.

Surveillance of adverse events revealed minimal gastrointestinal or other side effects (Extended Data Table 1). Adherence was equivalent between diets during the inpatient period (99.6±0.19% on MBD vs. 99.9±0.10% on WD, p = 0.27; Extended Data Fig. 2b). Next, we evaluated whether the consumed diets provided equivalent metabolizable energy, macronutrients, and the intended amounts of fiber and resistant starch. Analysis of intake during the nine inpatient days that provided meals exactly as designed (i.e., excluding the ad libitum feeding day and the gastric emptying test day which required a liquid meal) demonstrated that the diets consumed delivered the planned energy, macronutrients, and gut microbiome drivers (Extended Data Table 2).

Young, healthy, weight-stable individuals were enrolled to quantify whole body bioenergetics without the confounding effects of age and metabolic disease^14^, and to establish the comparative data needed for future studies enrolling people with various health conditions. The study sample was 30.8 ± 1.9 years of age, with a BMI within the normal weight to overweight range. All participants reported normal stool patterns based on the Bristol Stool Scale. We excluded people with recent antibiotic use or chronic health conditions by medical history and standard clinical labs (Extended Data Table 3).

## Diet modulated host metabolizable energy

**Fig 1.**
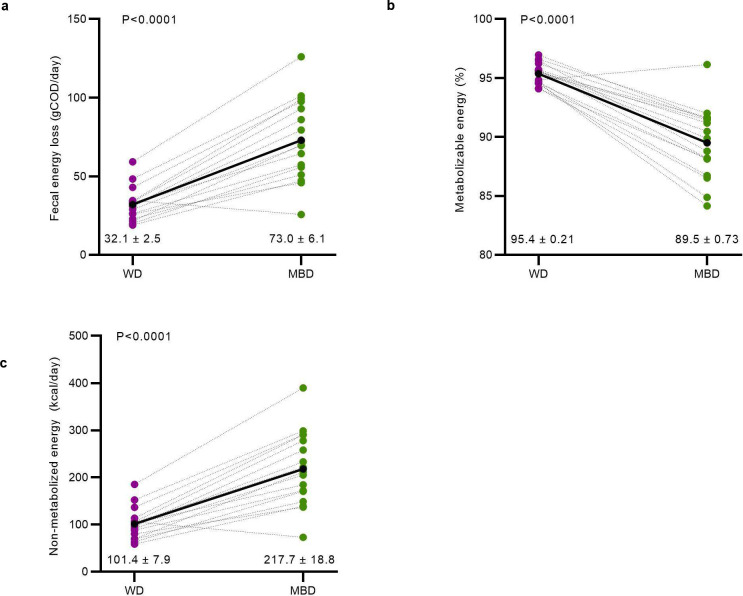
The microbiome enhancer diet reduced host energy harvest. **a**, Daily energy lost by each participant in feces on the WD vs. MBD in grams COD/day (gCOD/day). **b**, Host metabolizable energy based on the proportion of fecal COD to dietary intake. **c**, Calculated host non-metabolizable energy (kcals). All data reported as are mean ± s.e.m. N=17 per diet for all panels. COD—Chemical Oxygen Demand; MBD—Microbiome Enhancer Diet; WD—Western Diet

The overall goal of our study was to reprogram the gut microbiome and employ a quantitative paradigm with enough precision to detect within-participant responses to the diet intervention. To this end, as a more precise alternative to the use of ingested dyes, and according to the method of Pak^15^, we administered a low, non-laxative dose of non-absorbable non-digestible polyethylene glycol (PEG) with each meal. We measured the PEG concentration in fecal samples to normalize each fecal measurement to 24-hours based on expected daily PEG excretion. To quantify fecal energy loss, we used chemical oxygen demand (COD), a measure of electron equivalents in organic carbon^16^ and adjusted the result to PEG recovery. COD is typically used for microbial bioenergetics in environmental biotechnology^16^. We previously reported that, for food items, COD correlates highly to the commonly used bomb calorimetry method (R^2^=0.97)^17^. COD is a less expensive alternative that provides relevant information for microbial electron balances, and more physiologically relevant measurements, since COD does not include the oxidation of ammonia, which humans do not utilize as an electron donor^16,17^. Additionally, COD is advantageous because it simultaneously measures electrons available to humans and microbes, thus enabling electron balances to quantify energy flow^16^. Based on this (fecal energy as COD adjusted to PEG recovery), the MBD increased mean daily fecal energy losses, compared to the WD, over the six calorimetry days of the inpatient, controlled feeding period (73.0 ± 6.1 gCOD/day on MBD vs. 32.1 ± 2.5 gCOD/day on WD; P < 0.0001; Fig. 1a). When fecal energy loss was adjusted to total energy intake, host metabolizable energy was lower with the MBD (89.5 ± 0.73% on the MBD vs. 95.4 ± 0.21% on the WD (Fig. 1b; P < 0.0001), which equates to an additional 116 ± 56 kcals daily channeled to feces (Fig. 1c; P < 0.0001). These data align with the preclinical literature showing that the quantitative impact of the gut microbiome on host energy balance is primarily via its critical roles on energy harvest from the diet^8,9^.

## Diet reprogramed the gut microbiome

**Fig 2.**
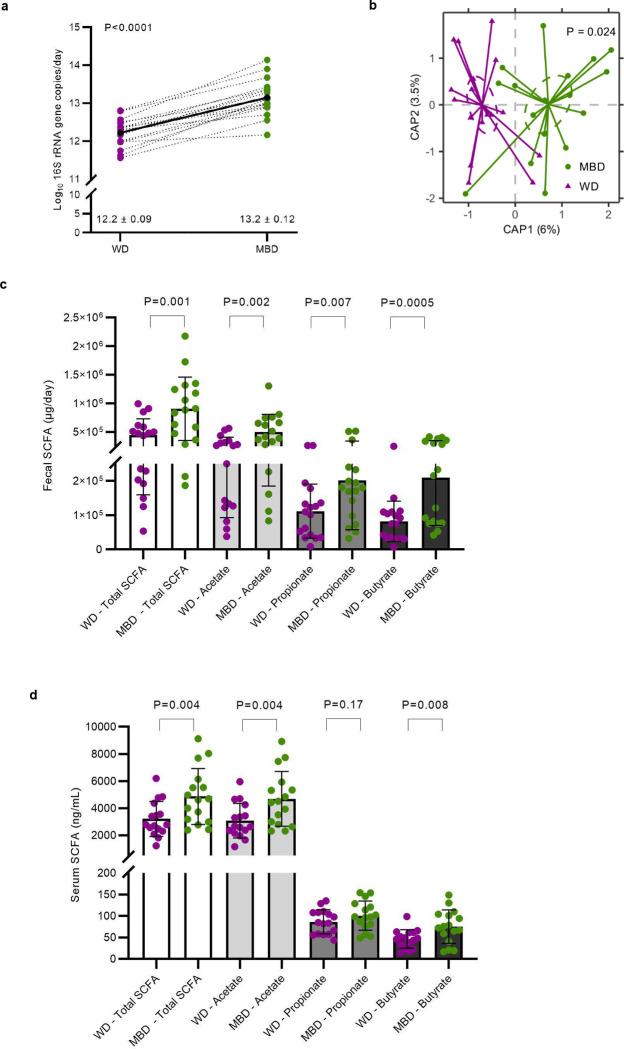

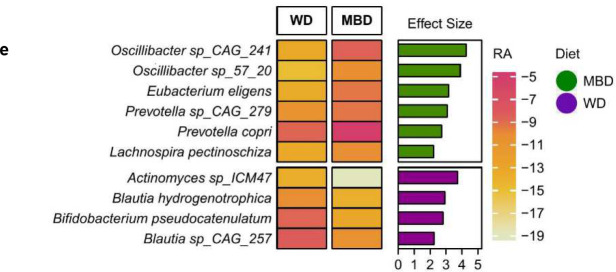
Diet reprogrammed the gut microbiome. **a**, Fecal bacterial biomass. **b**, Beta-diversity (Bray-Curtis Dissimilarity). **c-d,** Fecal and circulating short chain fatty acids. Data are presented as mean ± s.e.m for panels a-d. **e**, Heatmap shows the mean relative abundance of species whose relative abundance was significantly different by diet; bar plot shows the effect size of the regression coefficient from compound Poisson regression models comparing the relative abundance of each species by diet. Species shown in this figure were significantly different by diet (Q < 0.05), and the diet difference had an effect size ≥ 2. N=17 per diet for panels a-c and e; n=16 per diet for panel d. CAP—Canonical Analysis of Principal Coordinates; MBD—Microbiome Enhancer Diet; SCFA—short-chain fatty acids; WD—Western Diet

Given our primary finding that diet produced a clinically significant change in host metabolizable energy, we next evaluated the microbial phenotype associated with host energy balance. Mean daily fecal weight was higher on the MBD (P < 0.0001; Extended Data Fig. 3a), and a proportion of this additional weight was due to a significant increase in 16S rRNA genes (P < 0.0001; Fig. 2a), an indication of fecal bacterial biomass increase since the MBD produced 19.6 ± 3.5 gCOD/d of microbial biomass compared to 9.4 ± 1.2 gCOD/d on the WD.

Using whole-genome sequencing, we evaluated whether the increase in bacterial biomass was accompanied by a change in microbial diversity. Alpha-diversity assessed by bacterial richness and evenness did not differ (Extended Data Fig. 3b–c). In contrast, beta-diversity showed significant and stark separation by diet whether evaluated by Bray-Curtis (Dis)similarity (P = 0.02; Fig. 2b) or Jaccard Similarity (P = 0.02; Extended Data Fig. 3d). This diet-induced change in composition was paralleled by an increase in fermentation, evidenced by higher SCFAs on the MBD vs. WD in feces (total, acetate, propionate, and butyrate; P = 0.001, 0.002, 0.007, and 0.0005, respectively; Fig. 2c) and serum (total, acetate, and butyrate; P = 0.004, 0.004, and 0.008, respectively; Fig 2d).

To further explore the compositional changes in the microbiome associated with diet-induced changes in host metabolizable energy, we used metagenomic sequences to evaluate microbial taxonomic differences and derived regression coefficients describing each microbe’s association with diet using Maaslin2’s compound Poisson regression model^18^. Although relative abundance did not differ between the diets at the phylum and family levels (Extended Data Table 4), we found 53 differentially abundant taxa at the species level (P < 0.05; Extended Data Fig. 3e–f), of which 10 had a Q < 0.05 and differential effect size ≥ 2 (Fig. 2e). In accordance with dietary substrate availability, six species that had higher relative abundance on the MBD and included dietary fiber degraders *(Prevotella copri*, uncharacterized *Prevotella,* and *Lachnospira pectinoschiza*^19^; Q = 1.46 × 10^−06^, 0.0005, and 0.001, respectively) and/or butyrate producers (*Lachnospira pectinoschiza*^20^, *Eubacterium eligans*^21^, and likely the uncharacterized *Oscillibacter* (CAG_241 and 57_20)^22^; Q = 0.001, 7.44E × 10^−07^, 0.01 and 2.27 × 10^−07^, respectively). In contrast, the 4 species with a higher relative abundance on the WD included *Blautia hydrogenotrophica, Bifidobacterium pseudocatenulatum,* uncharacterized *Blautia CAG:257,* and uncharacterized *Actinomyces ICM7* (Q = 0.001, 5.6 × 10^−05^, 0.006, 0.02, respectively). These four species derive their source of fermentation from host-glycans, simple sugars^23,24^, or fermentation products generated by other gut microbes, mainly CO_2_^25^ and H_2_^26^. Thus, the microbiota signature that defined the response to the MBD i) channeled more energy to the microbes (instead of the host), ii) increased microbial fermentation, iii) increased fecal SFCAs, and iv) increased biomass. In contrast, the WD led to conditions in which the gut microbes were “starved” because a high proportion of metabolizable energy had been digested and absorbed by the host in the upper gastrointestinal tract.

## Host responded to microbiome reprogramming

**Fig 3.**
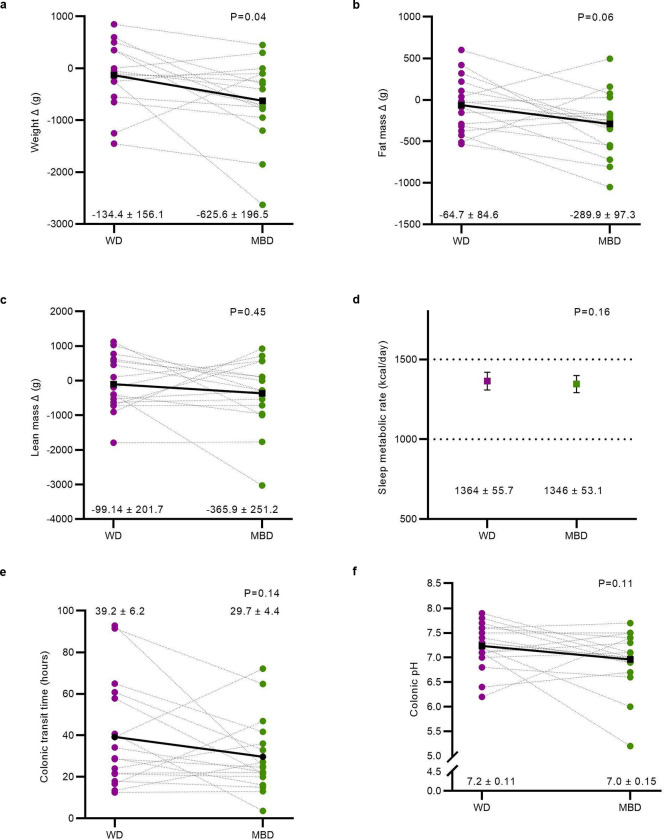

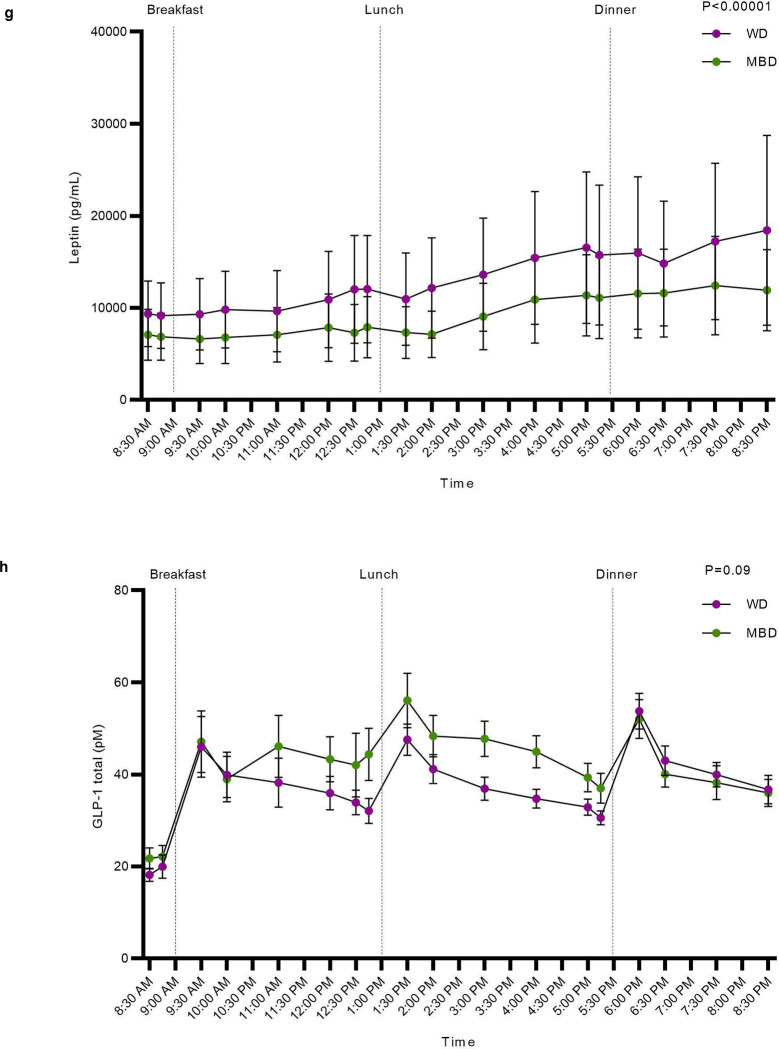

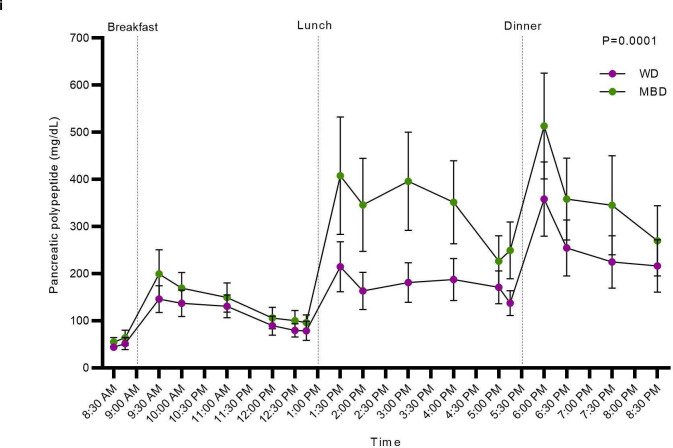
Host response to diet-induced gut microbiota reprogramming. **a**-**c**, Weight, fat mass and lean mass changes on the WD vs. MBD; n=16 per diet. **D**, Energy expenditure (sleep metabolic rate extrapolated to 24-hours); n=17 per diet. **E-f**, Colonic transit time and median colonic pH; n=17 per diet. **G-I**, An adipose-pancreas-gut appetite-modulating axis shown by leptin, GLP-1, and pancreatic polypeptide iAUC (n=15 per diet). All data reported as mean ± s.e.m. GLP-1—Glucagon-Like Peptide 1; iAUC—Incremental Area Under the Curve; MBD—Microbiome Enhancer Diet; WD—Western Diet

We explored whether the differential host energy harvest from the diet changed weight/body composition, gut motility, appetite, and/or hormonal secretion from the gut and pancreas. Although we previously showed that weight was stable within individuals during each inpatient calorimetry period, when either diet was consumed in random order^2^, we uncovered a small, clinically insignificant body weight reduction on both diets during the inpatient period, and the loss was greater on the MBD than on the WD (−134.4 ± 156.1 g WD; −625.6 ± 196.5 g MBD; P = 0.04; Fig. 3a). This change in weight was accompanied by a trend towards greater loss of fat mass on the MBD than on the WD (−64.7 ± 84.6 g WD; −289.9 ± 97.30 g MBD; P = 0.055) without a change in lean mass (−365.9 ± 251.2 g MBD; −99.14 ± 201.7 g WD; P = 0.45; Fig 3b–c). This suggests that the additional fecal-energy loss on the MBD was sufficient to promote a modest change in body composition despite equivalent metabolizable energy intake based strictly on existing food digestibility paradigms. These paradigms do not account specifically for the microbial biomass or microbial energy harvest^13^.

One of the gaps in prior human studies was the lack of a precise quantitation of the entire energy balance equation. In addition to our evaluation of energy intake (Extended Data Table 2) and fecal energy loss to derive host metabolizable energy (Fig. 1 a–c), we measured energy expenditure with whole room indirect calorimetry over 6 days and found no diet difference in sleep metabolic rate (in kcal/day) by diet (P = 0.15; Fig. 3d), despite being able to detect a posteriori a 26.5 kcal/day difference^2^. This suggests that, under conditions of fixed energy intake, the main quantitative contribution of the gut microbiome to host energy balance was through its effect on energy harvested from the diet, particularly when sufficient substrates were available for fermentation, as with the MBD.

The relationships among diet composition, gut microbes, and colonic transit time (CTT) are complex, multidirectional, and vary within individuals over time and between individuals^27^. Given the potential importance of CTT on the microbiota-driven host response to dietary manipulations, we evaluated whole-gut transit using a pH-sensing radiotransmitter device. We did not find a statistically significant difference in CTT by diet (39.2 ± 6.2 hours on WD vs. 29.7 ± 4.4 hours on MBD; P = 0.14; Fig. 3e). Gastric emptying evaluated by acetaminophen appearance in the blood after a fixed liquid meal also was not different by diet (Extended Data Fig. 4a). The pH of the colon can be an indicator of microbial fermentation activity. Neither median pH (which reflects both fermentation and the impact of food mixing in the colon) nor the median pH within a 1-hour window of the ileocecal passage (which is impacted primarily by microbial fermentation products)^28^ differed by diet (P = 0.11 and 0.23, respectively; Fig. 3f; Extended Data Fig 4b). The lack of statistically significant effects likely was due to the substantial amount of interindividual variability in CTT, gastric emptying and colonic pH, confirming the complex and individualized relationships among these parameters, which may be critical to understanding the host-microbiota axis within individuals^27^.

We hypothesized that the MBD might decrease appetite relative to the WD via the inclusion of high-fiber foods and production of metabolites through gut microbial fermentation^29^. This hypothesis was rejected (Extended Data Fig. 4c–h). Thus, the observed negative energy balance and small changes in body composition on the MBD did not trigger a compensatory change in appetitive behaviors or food intake compared to the WD.

The mammalian gut senses nutrients and microbial fermentation products and is part of the larger enteroendocrine system that plays a key role in maintenance of energy homeostasis^30^. Cumulative negative energy balances can result in body weight reductions. However, the regulation of body energy stores involves neural circuits in the hindbrain and hypothalamus, proximal and distal gut hormone secretions and adipose tissue neural and endocrine signals to the brain^31^. We explored several potential mechanisms by which the gut microbiome might regulate body weight beyond the observed negative energy balance. On the second-to-last day of each inpatient period, we measured fasting and postprandial levels of several hormones known to regulate appetite at 18 timepoints over 12-hours. Consistent with the slight, but measurable decrease in body fat stores on the MBD, secretion of the adipose tissue hormone leptin had a significantly lower incremental area under the curve (iAUC) on the MBD (P = <0.0001; Fig. 3g). A reduction in circulating leptin is known to increase food intake^32^. GLP-1 is a gut incretin hormone secreted by L-cells in the proximal gut in response to meals. GLP-1 is also secreted from the distal colon in response to gut microbiome metabolites including SCFA^29^. The increase in fecal and serum SCFA on the MBD was accompanied by a trend of increased GLP-1 iAUC (P = 0.08; Fig 3h), with a significantly higher AUC at breakfast and lunch and a trend towards a higher AUC at dinner (P = 0.02, 0.04 and 0.08, respectively) on the MBD compared with the WD. Pancreatic Polypeptide (PP) iAUC was significantly increased on the MBD (Fig 3i). GLP-1 and PP decrease food intake^33^. Therefore, the short-term negative energy balance within our experimental paradigm did not trigger the compensatory food-intake responses expected from the change in body fat and leptin. Further experiments should pursue this hypothesis.

## Microbes contributed to energy balance

Given the robust response to our diet intervention by the gut microbiome and host, we sought to determine the quantitative role of the gut microbiome on energy harvest from the diet versus the impact driven solely by food digestibility^11^. We tested the hypothesis that methane production by methanogenic archaea contributes to a net negative energy balance. We developed and validated a first-in-human method to quantify 24-hour methane production in a whole room calorimeter at part-per-billion resolution^34^. The range of methane measured within our study was 0.28–1613 ml/day, translating to 0.002–14 kcals lost per day. While this negative energy balance is not clinically meaningful for weight management by itself, it may have a quantitative impact when considered in concert with other microbial impacts on host energy balance. For example, methanogenesis might accelerate fermentation based on removing a thermodynamic limitation or by competing with other hydrogen-consuming process, like acetogenesis and sulfate reduction^35^.

Host metabolizable energy on the WD showed little interindividual variability (94.1–97.0%; Fig. 1b) since most nutrients were absorbed in the small intestine and were inaccessible to the gut microbiome. However, the range of host metabolizable energy in response to the MBD was much broader (84.2–96.1%; Fig. 1b). The range translates to 69–408 non-metabolized kcals/day (vs. 56–185 kcals/day on the WD), a clinically meaningful quantitative difference that could tip the scale towards a greater negative energy balance based on dietary energy harvest efficiency.

**Fig. 4.**
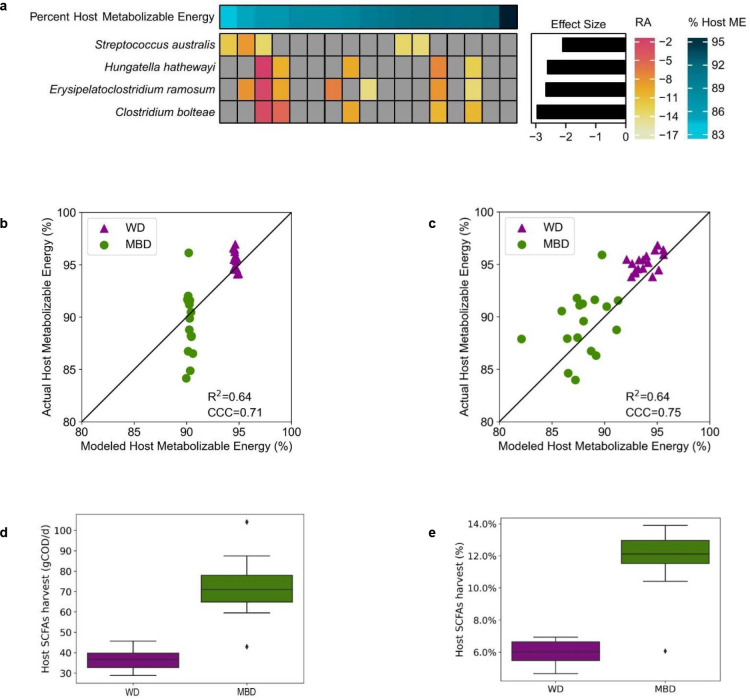
The contributions of the gut microbiome to host energy harvest. **a,** The heatmap shows the associations between host ME and the mean species RA. Each row is a microbial species and each column is an individual participant. The bar graph shows the effect size of the regression coefficient between the independent variable of host metabolizable energy and each species, from compound Poisson regression models. Figure shows all significant associations with Q < 0.05 and effect size ≤ 2. **b**, An in silico model comparison of modeled host ME vs. actual ME using the same fixed CTT (48 h) for all participants. **c,** The same model with each participant’s measured CTT. **d**, Box plot shows microbial energy harvest through SCFAs as grams COD per day (gCOD/d) for the WD and the MBD. gCOD were calculated as the sum of acetate, propionate, n-butyrate, and iso-butyrate absorbed. Data reported as median with error bars showing minimum and maximum values and box ends showing the 2^nd^ and 3^rd^ quartiles. Diamonds are outliers that fall outside 1.5X IQR. **e**, The percentage of COD absorbed as SCFAs adjusted for total energy intake (in gCOD/day). N=17 per diet for all panels. CCC—concordance correlation coefficient; COD—Chemical Oxygen Demand; CTT—Colonic Transit Time; Host ME—Host Metabolizable Energy; IQR—Interquartile Range; MBD—Microbiome Enhancer Diet; SCFA—short-chain fatty acids; RA—Relative Abundance; WD—Western Diet

This led us to hypothesize that the variability in host energy balance could be associated with the repertoire of gut microbes in the colon. To test this hypothesis, we asked whether the quantitatively important variability in host metabolizable energy on the MBD could be related to a unique microbial signature. To identify those microbial signatures, we derived regression coefficients describing each microbe’s association with the independent variable of host metabolizable energy using Maaslin2’s compound Poisson regression model^18^. In total, host metabolizable energy was associated with 16 species (Extended Data Fig. 5a–b). The significant microbes with the largest effect size (Q < 0.05; effect size ≥ 2) were *Clostridium bolteae*, *Streptococcus parasanguinis*, *Streptococcus australis*, and *Erysipelatoclostridium ramosum*. All were inversely associated with host metabolizable energy, indicating that reduced energy availability to the host may increase substrate availability for the growth of these specific microbes (Fig. 4a).

We next embarked on a series of mathematical modeling experiments using an established model^36^ to estimate the gut microbial contribution to host energy balance. We previously reported an in-silico model that estimates the dual impact of host digestion and microbial fermentation on macronutrient uptake in the small and large intestine and ultimately, on host metabolizable energy^36^. The model also estimates the amount of SCFAs absorbed by the host due to microbial fermentation in the colon and the associated biomass. We applied this model to predict the host metabolizable energy we measured in our study by inputting actual energy intake components and fecal energy in grams COD/day. Our previously published model used a fixed CTT of 48 hours, which is a reasonable population-level estimate for healthy adults^37^. With a fixed CTT, the modeled host metabolizable energy for participants on the WD was 95.2 ± 0.001% and for MBD was 92.4 ± 0.001% (Fig. 4b). This is similar to the mean host metabolizable energy we measured on the WD and the MBD (95.4 ± 0.21% and 89.5 ± 0.73%, respectively; Fig. 1b). However, the variability we saw experimentally on the MBD was not reproduced by the mathematical model. We hypothesized that we could improve the model’s predictive ability by adding measured CTT since it is a key modulator of microbial composition, fermentation, and host energy balance^27^. When we included measured CTT, the modeled range of metabolizable energy on the MBD was 84.6–92.9%, which was very similar to the measured range of 84.2–96.1%; furthermore, systematic and proportional bias was minimized (Extended Data Fig. 5c–d). Thus, using the CTT explained some of the variability in host metabolizable energy.

A significant proportion of the reduced metabolizable energy on high-fiber diets is due to colonic microbial fermentation of fiber and resistant starch into absorbable SCFA^38^. Our model predicted that more total energy (g COD) as SCFAs was absorbed by the host on the MBD, compared to the WD (72.3 ± 13 gCOD/d on the MBD vs. 36.4 ± 4.3 gCOD/d of microbially-derived SCFAs; P < 0.00001; Fig. 4d). When we adjusted the SCFA absorption for energy intake, we found a nearly 2-fold greater absorption of energy as SCFAs on the MBD as compared to the WD (P < 0.00001; Fig. 4e). Therefore, despite less total energy being absorbed by the host on the MBD, a larger proportion was derived from SCFAs. Consistent with our experimental data, our model strongly supports a significant microbial contribution to host metabolizable energy and, therefore, the overall energy balance.

## Discussion

Microbial communities in the gut have a profound impact on mammalian host endocrinology, physiology, and energy balance, with most causal inferences historically restricted to preclinical animal models^1,8,9^. Prior human studies exploring the relationships among the gut microbiome, obesity, and energy balance lacked the deep phenotyping, precise methodologies, and rigorous controls that are instrumental for drawing causal inferences with respect to human health. Our central finding was that a diet designed to feed and reprogram the colonic gut microbiome, under conditions of fixed energy intake and physical activity, led to reduced metabolizable energy by the host and increased fecal and energy output consisting of undigested food, bacterial biomass, and microbial metabolites. Thus, the greater fecal energy loss on the MBD was not just due to undigested food, but also to an in increase in fermenting gut microbes and their metabolites. The direction of change in energy absorbed from the diet by the host (lower on the MBD) was consistent in 16 out of 17 study participants. This means that most of our participants had the microbial capacity to metabolize dietary substrates. This observation is contrary to the “extinction” hypothesis proposed in mice^39^ and suggests that, with rigorously controlled dietary conditions that vary markedly in the amount of substrate delivered to the colon with a comparable kcal and total macronutrient profile, healthy humans harbor microbiomes which are adaptable and/or have sufficient functional redundancy to overcome certain extinctions imposed by diet.

The reduction in energy harvest from the diet on the MBD relative to the WD was not accompanied by a reduction in energy expenditure or an increase in hunger or ad libitum energy intake. However, the significant diet-induced reprogramming of the gut microbiome was accompanied by a modest change in weight and body composition with robust enteroendocrine signals from the adipose-pancreas-gut axis. Given the modestly negative energy balance and reduced body energy stores on the MBD, our results support the hypothesis that an intentional remodeling of the gut microbiome through provision of adequate dietary fiber, resistant starch, and a focus on whole, minimally processed foods resets the integrated sensing mechanisms known to affect food intake and body energy stores. One or more of these mechanisms or other unknown mechanisms might be responsible for the population associations between a diverse human gut microbiome and lower body mass^1^. The slightly greater reduction in weight and body fat on the MBD, compared to the WD, over the inpatient period despite daily titration of energy requirements to match calorimetry-derived measures of energy expenditure, suggests that the use of a diet that adequately feeds colonic microbes and increases microbial fermentation products (i.e., short-chain fatty acids) will not lead to additional absolute energy availability to the host. In contrast, diets such as the MBD promote additional fecal energy loss and an increase in host uptake of SCFAs from the colon, despite the overall decrease in host uptake of energy. Future microbiome-focused research should delve into these systems for controlling body weight.

The quantitative contributions of gut microbes to host energy balance were addressed in two forms. First, the energy in feces increased by 40.9 ± 4.6 g COD/d (116 ± 56 kcals kcal/day) for participants on the MBD, even though their total metabolizable energy intake was the same. Second, the microbial community increased in size (biomass) and fermentation processes that were reflected by increased fecal and serum SCFAs on the MBD as compared to the WD. Thus, the host’s energy intake shifted towards microbially produced SCFAs and away from proximally digested and absorbed carbohydrates in the food. While the quantitative contribution of microbially generated SCFAs was overshadowed by the additional loss of microbial biomass in the feces, the uptake of more microbially produced SCFAs was associated with increased GLP-1 and pancreatic polypeptide concentrations.

We also found a taxonomic signature that was in alignment with the expected impacts of the substrates available to the gut microbes on the two diets. First, many of the species detected at higher abundance on the MBD were fiber degraders and/or butyrate producers. Second, our data reveal that, when the gut microbiome was reprogrammed by providing adequate dietary substrates, changes in the relative abundance of microbes that produce SCFAs could modulate several components of the energy balance equation. *Lachnospira pectinoschiza*, *Eubacterium eligans*, and likely the uncharacterized *Oscillibacter* had a higher relative abundance on the MBD and are butyrate producers^20–22^. Butyrate plays important roles in host energy balance by stimulating the release of satiety hormones^40^ and accelerating CTT^41^. Acetate stimulates the release of satiety hormones^42^ and acts as a satiety signal itself ^43^.

Host metabolizable energy was highly variable on the MBD. Given our tight control of energy intake and energy expenditure, this suggests that the microbial contribution to this variability was greater in some hosts than others. Indeed, with a proportionally equivalent input of substrates for microbes, fecal energy losses varied over an ~6-fold range. Understanding the mechanisms by which the microbial communities in the human colon modulate energy harvest and their interaction with host factors such as CTT will provide valuable quantitative data to drive personalized strategies to optimize host-microbiota-diet interactions and prevent or treat obesity.

Host metabolizable energy was associated with a unique microbial profile on the MBD, with 4 microbial species whose relative abundance increased in association with decreasing host metabolizable energy. One of those species, *Streptococcus australis*, transiently increases after weight loss due to bariatric surgery as compared to normal weight controls^44^. *Hungatella hathewayi* and *Erysipelatoclostridium ramosum* were more abundant in germ-free mice colonized with feces from a human that underwent caloric restriction with a concomitant phenotype characterized by lower adiposity^45^. *Clostridium bolteae*, in addition to being a lactic-acid producing bacterium^46^, has recently been reported to bind phenylalanine, tyrosine, or leucine amino acids to microbially deconjugated bile acids. While the clinical effects of these microbially transformed bile acids are unclear, bile acids are known to play an important role in microbial energy extraction^47^. Overall, these findings make it plausible that the variability in host metabolizable energy on the MBD is related to a specific microbial signature and the metabolic processes driven by the relationships between host and microbes.

We also investigated, in silico, the factors that might be contributing to hose metabolizable energy variability and found that colonic transit time was an important driver. Host metabolizable energy prediction with measured CTT more closely captures the variability seen in measured host metabolizable energy. Our mathematical model, which generated outputs consistent with the clinical data describing the metabolizable energy of participants consuming WD and MBD, allowed us to determine the important role of CTT and to quantify that a host on the MBD produced feces containing 19.6 ± 3.5 gCOD/day of microbial biomass (about 10 gCOD/day more than WD) and led to 36.4 ± 4.3 gCOD/day more uptake of microbial derived SCFAs. We believe these factors, and others that may be revealed in future studies, could capitalize on the adaptability of the gut microbiome as a target for personalized medicine^48^.

Given the size and scope of the global obesity epidemic and its continued increase, new solutions are needed. The scientific community has recently reoriented itself towards population interventions that promote small changes in energy intake and expenditure as a means of preventing weight gain^49^. This study demonstrates the potential to enact this “small changes” principle through the consumption of a simple whole food intervention which reprograms the gut microbiome and might serve as a useful population level tool to fight the global obesity epidemic.

## Methods

The design of this trial (NCT02939703) has been previously reported^1^. We summarize key elements below and include details on elements not reported elsewhere.

### Study participants.

We recruited males and females 18–45 years of age with a BMI ≤30 kg/m^2^ who were weight stable and otherwise healthy after obtaining informed consent^1^. The study was approved by the AdventHealth Institutional Review board. Adverse events were monitored at each contact with the participant and reported according to Institutional Review Board guidelines.

### Design overview.

This was a randomized crossover study with a control Western Diet (WD) compared to a Microbiome Enhancer Diet (MBD). After an initial assessment period to establish outpatient dietary intake requirements (Days 1–9), all food was provided to participants, and they consumed the meals outpatient for 11 days (Days 10–20 and 39–49) and inpatient for 12 days (Days 21–32 and 50–61). Included was a minimum 14-day washout between diet periods. All endpoint assessments were conducted while participants were housed in our metabolic ward^1^.

### Clinical Assessments.

Health status was determined by medical history, physical examination, standard blood chemistries, and the Bristol Stool Scale to evaluate stool type based on shape and consistency with scores of 3–4, indicating neither constipation nor loose stools^2^.

### Whole-room indirect calorimetry.

Energy expenditure and all its subcomponents was evaluated every 24-hours with whole room calorimetry in two 6-day blocks per diet (days 24–29 and 53–58^1^) following published standards of operation^3^. We also re-engineered our calorimeters to measure 24-hour methane. The full validation of this method has been previously described^4^. Activity was tightly controlled during the day to maintain spontaneous physical activity consistent within and between participants^1^.

### Energy balance.

Energy balance was estimated by subtracting actual metabolizable energy intake (calculated by menu design software based on actual intake) from energy expenditure measured by whole room calorimetry^5^.

### Host Metabolizable Energy.

To calculate host metabolizable energy, we converted energy intake in kcals to grams COD using our published relationship^6^. That allowed us to compute the percent of energy metabolized by the host after accounting for fecal energy loss which was measured in COD. To relate this percentage back to kcals and determine the number of daily kcals that were not absorbed by the host, we multiplied host metabolizable energy percentage by energy intake in kcals.

### Dietary intervention.

Diets were prepared in our metabolic kitchen based on kcals needed to maintain energy balance as determined by whole-room indirect calorimetry. Diets were designed with menu software (ProNutra Version 3.5, Viocare, Inc, Princeton, NJ) that proportionately calculated diets based on energy needs. Duplicate meals were prepared during all calorimetry days and evaluated for energy content as a quality-control step (Eurofins, Madison, WI). Nutritional composition of the diets was based on the menu software database (USDA Database Standard Reference 23), with the exception of resistant starch, because it is absent from all currently available nutritional databases. We limited foods containing resistant starch on the WD and then estimated the content on both diets based on published estimates of resistant starch content of common foods^7^. Diets were equivalent in metabolizable energy and proportions of macronutrients. As much as possible, we used similar types of foods on both diets to minimize differences in micronutrients. Extended Data Table 2 shows the energy, macronutrient, and drivers in each diet. Consumption of 100% of provided foods was required. Diet adherence was monitored during the 11-day outpatient phase at clinic visits 2 or 3 times per week where at least one meal was consumed on site. During the inpatient phase, all meals were monitored^1^.

### Sample collection, processing and shipment.

Blood, urine, and fecal samples were collected in our metabolic unit using standard protocols. Fecal samples were collected each time they were produced during the 6-day measurement periods in the whole room calorimeter. Samples weight were tracked upon collection and as aliquots were prepared. Fecal samples were processed within an hour of production under an anaerobic hood and were maintained on ice during processing. After mixing with a sterile spatula, samples were sub-aliquoted for various downstream applications. Samples for metagenomic sequencing were snap frozen without additives and stored at −80°C. They were shipped overnight on dry ice. Any fecal sample not needed for method-specific aliquots were stored (sealed) in original collection container at −20°C within 60 minutes of collection. At the end of each 6-day calorimetry period, all collection containers were opened, and all frozen samples were transferred into a single, large, homogenization container to create a composite sample (without additives) that was used to measure fecal energy, SCFAs, and biomass. The composite was partially thawed on ice while remaining sealed and then homogenize, on ice, using a sterile paddle homogenizer. The composite sample was stored at −80°C until used or shipped overnight on dry ice^1^.

### Weight and body composition.

Weight (fasting and in a gown) was measured daily during the 12-day metabolic ward stay on a calibrated scale. Body composition was assessed with dual energy x-ray absorptiometry the day prior to entering the calorimeter (Days 23 and 52) and after exiting the calorimeter (Days 31 and 60) with a two-day window allowed for the pre or post measurement.

### Fecal energy.

Fecal energy was evaluated with chemical oxygen demand (COD) as per our previous publication^6^. Briefly, COD was measured per manufacturer’s protocol using a reactor digestion method with high-range digestion vials followed by a colorimetric assay (HACH, Loveland, CO; Product # 2125925). To ensure that fecal energy was accurately reflective of 24-hour fecal production, we utilized the non-absorbable, non-digestible fecal marker polyethylene glycol (PEG). Participants consumed 1.5g/day of PEG of molecular weight 3350 g/mol (PEG3350). The PEG3350 was procured by a compounding pharmacy that prepared 0.5g capsules (percent error = 2.8%) (Pharmacy Specialists, Altamonte Springs, FL). The details of the PEG assay are below. Fecal energy was measured in 6-day composites of feces collected in our calorimeters. We normalized fecal energy produced to the weight of all feces produced in those 6-days and then to PEG recovery. Fecal energy loss was converted to host metabolizable energy by calculating the percentage of energy that was lost in feces (in g COD) relative to total energy intake (in g COD). The conversion from energy in COD to kcals lost in feces per day (non-metabolizable kcals) was calculated by multiplying total EI in kcals by the percent host metabolizable energy.

### Polyethylene glycol assay.

We utilized a method that is slightly modified from the initial published method by Sadilek et al.^8^. Key modifications include quantitation based on the +2 charged PEG3350 polymers instead of the +4 charged polymers and the inclusion of an internal standard. Sample preparation was also slightly modified. Briefly, samples were prepared by a 1:1 dilution with Nanopure water and homogenized. Two grams of sample was diluted in 14 ml Nanopure water that included a final concentration of 1.5 uM internal standard (monodispersed PEG, MW 2160 g/mol; Quanta Biodesign, Plain City, OH; Product # 10897). An HPLC-MS method was used for the separation and detection of PEG3350 in human fecal samples^8^. The published assay was transferred to ARL Biopharma for subsequent PEG quantification on study samples (Oklahoma City, OK). The assay is linear as evidenced by the R^2^ of the calibration curve (0.9987). The linear range of the assay was from 0.1 uM to 20 uM with PEG3350 recovery ranging from 96.2–104.5%. The relative standard deviation of the assay was 1.8%. There was no co-elution of analyte with expected excipients or related compounds in chromatograms demonstrating the assay is specific for PEG3350.

### Quantification of bacterial 16S rRNA genes.

Quantitative PCR (qPCR) was performed with triplicate PCR reactions as described by^9^ in a Thermofisher Applied Biosystems Quant Studio 3. Universal primers 926F (5’ – AAACTCAAAKGAATTGACGG - 3’) and 1062R (5’ - CTCACRRCACGAGCTGAC - 3’) were used. Calibration curves using 7 data points were generated on each run using plasmids with 16S rRNA genes, and adding a plasmid concentration to achieve copy numbers in the range from 10^1^ to 10^9^ per reaction. Reaction mixtures with a final volume of 20 μL, comprised of10 μL 2 × Fast-Start SYBR green, 0.6 μL each forward and reverse primer (final concentration, 0.3 μM), 2 μL DNA template (equilibrated to 10 ng), and DIH_2_O to 20 μL. Themocycler conditions were 95°C for 5 minutes, followed by 30 cycles of 95°C for 15 seconds, 61.5°C for 15 seconds, and 72°C for 20 seconds, and a final elongation step at 72°C for 5 minutes. Standards were made by cloning the *E. coli* 16S rRNA gene using the Thermofischer TOPO TA Cloning Kit. Plasmids were purified using the Qiagen QIAprep Spin Miniprep Kit. Purified plasmids were quantified by Qubit. Plasmid copy number was then calculated using the following formula:

$$\;Copy\;Number\;=\frac{\left[DNA\left(\frac{ng}{\mu L}\right)\right]\;*\;6.022\;*\;10^{23}}{Plasmid\;legnth\;(bp)\;*\;10^{9}\;*\;660}$$

16S rRNA gene copy numbers per gram of feces were used to calculate daily copy numbers by multiplying by fecal weight and adjusting to PEG recovery.

### DNA and RNA sequencing.

Fecal sample processing, nucleic acid extraction, library preparation, and sequencing were performed at the University of North Carolina at Chapel Hill Microbiome Core, which is supported by the following grants: Gastrointestinal Biology and Disease (CGIBD P30 DK034987) and the UNC Nutrition Obesity Research Center (NORC P30 DK056350. DNA was extracted using the QIAamp Fast DNA Stool Mini Kit and library was prepared using the Swift 2 S Turbo DNA library kit. RNA was extracted using the Qiagen RNeasy PowerMicrobiome kit and library was prepared using QIAseq Stranded Total RNA Library kit. DNA and RNA were sequenced on the Illumina HiSeq 4000 PE 150 platform. Mean total reads were 18,339,758, with similar read depth on each diet (19,475,004 for the WD and 17,204,513 for the MBD).

### DNA Sequence Processing.

DNA and RNA sequencing output was quality controlled with FastQC^10^. Adapters were trimmed using TrimGalore^11^. DNA sequences were aligned to Hg38 using bowtie2^12^ and RNA sequences were aligned to Hg38 using STAR^13^. DNA and RNA sequences were then analyzed for taxonomic composition with MetaPhlAn3^14^, using standard parameters.

### Species Alpha- and Beta-Diversity.

All calculations and analyses were conducted in R^15^. Taxonomic composition output from MetaPhlAn3 was processed for beta-diversity analysis using the “phyloseq” R package^16^. A rarefaction curve was created using the “vegan” R package^17^ to determine the optimal count-depth for rarefaction. Once the optimal count-depth was determined, rarefaction was performed using phyloseq. Alpha-diversity metrics were calculated using the “microbiome” R package^18^. After samples were rarified, each sample had 3,578,445 sequences. Bray-Curtis and Jaccard distance matrices were calculated on the rarefied count data using vegan. The distance matrices were tested for significance by PERMANOVA using vegan. Beta-dispersion was calculated, and the results tested for significance with the ANOVA-like permutation test and Tukey’s HSD in the vegan. Constrained Analysis of Principal Coordinates (CAP) ordination was performed with vegan. Statistical significance testing was performed with PERMANOVA in base R. Beta-diversity ordination figures were created using the “ggplot2” R package^19^. Differential abundance heatmap figures were created using the “ComplexHeatmap” R package^20^.

### Differential Abundance.

Differential abundance testing by diet and host metabolizable energy was carried out using the output of MetaPhlAn3 in the “MaAsLin2” R package^21^. Taxonomic counts were filtered with a 25% prevalence cut-off. Compound Poisson multivariate linear models were used to account for zero-inflated data^21^. In the diet analysis the dependent variable was microbial abundance, the fixed variables were diet, period, and period sequence, and participant ID was a random factor. In the host metabolizable energy analysis, the dependent variable was microbial abundance, and the fixed independent variable was host metabolizable energy.

### Short-chain fatty acids.

A targeted short chain fatty acid (SCFA) panel including acetate, propionate and butyrate was conducted for both fecal and serum SCFA (Metabolon, Inc., Mooresville, NC). For fecal SCFAs, the concentrations were adjusted for total feces produced and PEG recovery to calculate the total fecal SCFAs over the 6 inpatient calorimetry days. Acetate, butyrate, and propionate were summed to calculate total fecal SCFA and total serum SCFA.

### Appetite.

Subjective ratings of appetite were determined using visual analog scales (VAS) administered at −30, −15, +30, +60, +120, and +180 min pre/post each meal. Breakfast was fixed at 500 kcals and lunch and dinner provided 1.5 X the energy content of their energy balanced diet consumed while in the whole room calorimeter, which is equivalent to 1.3X the energy needed in free-living conditions on our metabolic ward. Ad libitum intake was allowed at lunch and dinner for assessment of changes in food intake^1^. The trapezoidal rule was used to calculate the iAUC per meal and diet for each appetite scale^22^.

### Gut transit time.

A radiotransmitter motility capsule was used to determine transit time and pH in the colon (SmartPill^™^; Medtronic, Minneapolis, MN)^1,23^.

### Gastric emptying.

Gastric emptying was assessed via acetaminophen appearance after a test meal. Acetaminophen (1,500 mg) was administered at nominal timepoint zero^1^.

### Enteroendocrine hormones.

Enteroendocrine hormones were evaluated after a test meal (Boost Plus or equivalent, 500 kcal) and lunch/dinner from their assigned diet at nominal timepoints −30, −15, + 30, +60, + 120, and +180 minutes pre/post each meal^1^. GLP-1 (active), Leptin, and Pancreatic Polypeptide were measured with V-PLEX Metabolic Panel 1 Human Kit (MesoScale Diagnostics, Rockville, MD; Product # K15325D). For enteroendocrine hormones, the iAUC for the total time of measurement (~11 hours) was calculated by diet. The trapezoidal rule was used to calculate the iAUC^24^.

### Mathematical modeling.

Previously, we developed a multicompartment transit, reaction, and absorption model with these 3 compartments: upper gastrointestinal tract, lower gastrointestinal tract, and the remaining human body. The code for the model can be found here: https://github.com/amarcus1/Metabolizable-and-digestible-energy-calculator-for-patients-with-small-intestine-removed. The model estimates human dietary absorption for a general population and humans who had sections of small intestines and large intestines surgically removed. Specially, the model calculates the host harvest of carbohydrates, protein, and fat in the upper GI and microbe-derived SCFAs in the lower GI^25^. For each participant, we had daily and cumulative values for grams of carbohydrates, proteins, fat, total fiber, and resistant starch consumed based on our designed menus. To use this information in our mathematical model, we systematically converted the measurements into gCOD/day of a) Available Sugar and Starch; CHO (g) - Resistant Starch (g) – fibers (g), b) Resistant Starch (RS), c) Non-Starch Polysaccharides (NSP), d) Proteins, and e) Fat. These data were input into the model to calculate host metabolizable energy and compare it to our measured data. We then improved the model by using the measured colonic transit time and evaluated the impact of this change by comparing actual versus modeled data and calculating the coefficient of determination and concordance correlation coefficient. We evaluated systematic and proportional bias with a Bland-Altman plot^26^. We compared the absolute and proportional SCFA absorption with a paired two-tailed t-test.

### Statistical analyses.

Descriptive statistics for continuous variables are presented as mean ± standard error of the mean if normally distributed or as median (interquartile range) if non-normally distributed; categorical variables are shown as counts and percentages.

Appropriate to our randomized crossover design, we used a linear mixed model (SAS PROC MIXED) with diet, period, and sequence as fixed effects and participant as a random effect to compare differences by diet in our primary endpoint (host metabolizable energy: fecal energy loss adjusted to energy intake) and most other secondary and exploratory endpoints. When the distribution of the model residuals was found to deviate considerably from normality, a logarithmic transformation was applied. For each endpoint, we included only participants with complete data for both diet interventions when the data were considered to be missing not at random. Several values that were considered to be missing at random for the enteroendocrine hormone data (i.e., due to temporary issues with blood draw or laboratory analysis, but not because the entire sample was missing) were imputed by carrying the last observation forward or using the interpolation method (i.e., averaging the previous and subsequent values) ^27^. Statistical analyses were performed using SAS 9.4 and R.

A p-value less than 0.05 was considered statistically significant. When using the false discovery rate (FDR)^28^ to correct for multiple comparisons for differential abundance analysis of gut microbial composition and associations of gut microbes with host metabolizable energy, an FDR q-value <0.05 was considered statistically significant.

## Extended Data

**Extended Data Fig. 1.**
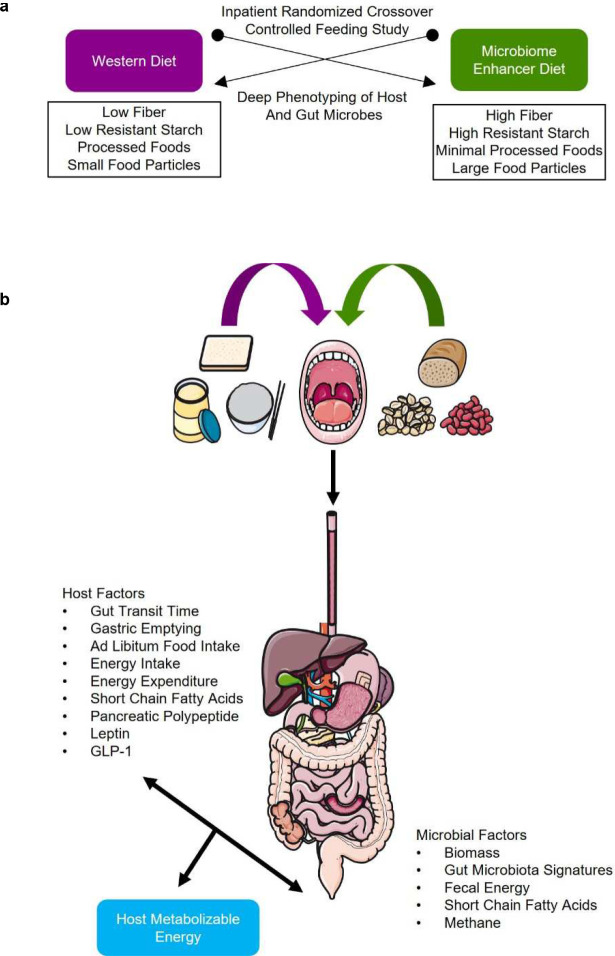

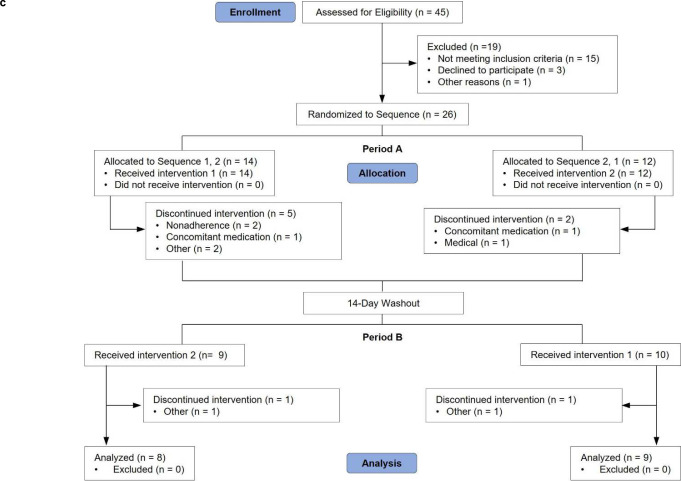
Summary of results and study flow. **a,** Schematic of overall study design. **b,** Summary of key microbiome and host factors that are collectively associated with host metabolizable energy in our study. **c,** CONSORT diagram showing the flow of participants from enrollment through analysis.

**Extended Data Fig. 2.**
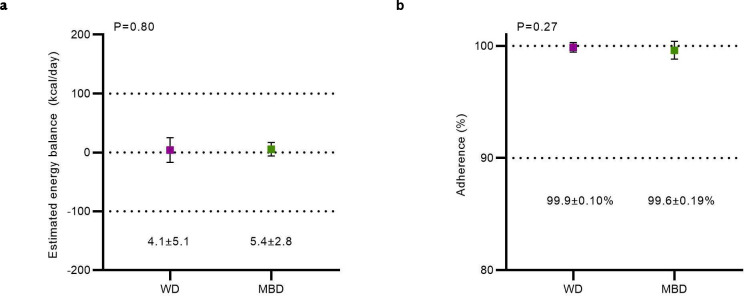
The experimental paradigm achieved adherence and energy balance. **a,** Energy balance (mean of 6 measurement days) estimated from traditional parameters: Energy Balance = Energy Intake (kcals/24h) – Energy Expenditure (kcals/24h). **b,** Dietary adherence on the WD compared to the MBD over all inpatient days where all 3 meals were consumed on-site, and no changes were made to the feeding for testing. All data reported as mean ± s.e.m. N=17 per diet for both panels. MBD—Microbiome Enhancer Diet; WD—Western Diet.

**Extended Data Fig. 3.**
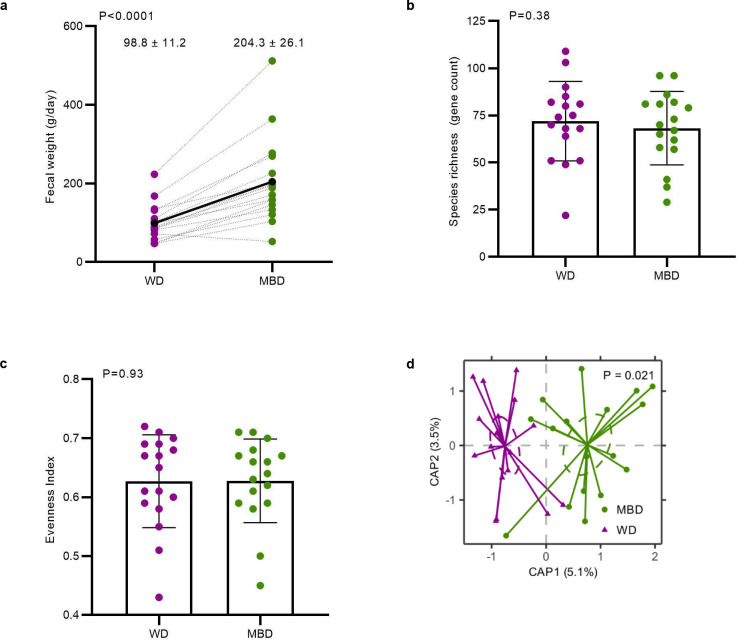

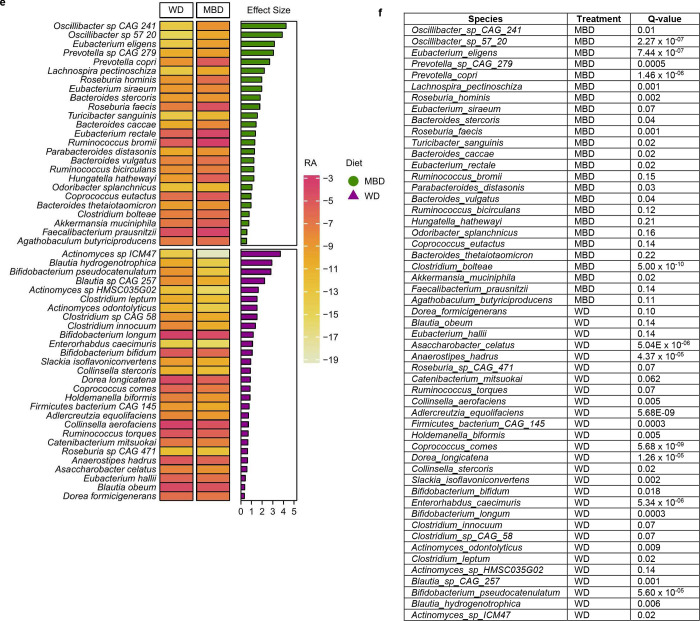
Gut microbiome community structure and reprogramming. **a,** Fecal weight from 6 days of composited feces averaged to generate daily production. Data reported as mean ± s.e.m. **b-c,** Alpha-diversity measures of richness and evenness. **d,** Beta-diversity at the species level assessed with Jaccard Similarity. **e,** Mean relative abundances and effect sizes of significantly differentially abundant species between diets. The heatmap shows the mean relative abundance of significant species between each diet. The bar graph shows the effect size of the regression coefficient for the comparison of species relative abundance by diet. Species shown in this figure had P < 0.05 and Q < 0.25. **f,** Q-values for each regression coefficient shown in **e**, which ranged from 5 × 10^−10^ to 0.217. The treatment indicates the diet on which the species relative abundance was higher. N=17 per diet for all panels. CAP—Canonical Analysis of Principal Coordinates; MBD—Microbiome Enhancer Diet; RA—relative abundance; WD—Western Diet

**Extended Data Fig. 4.**
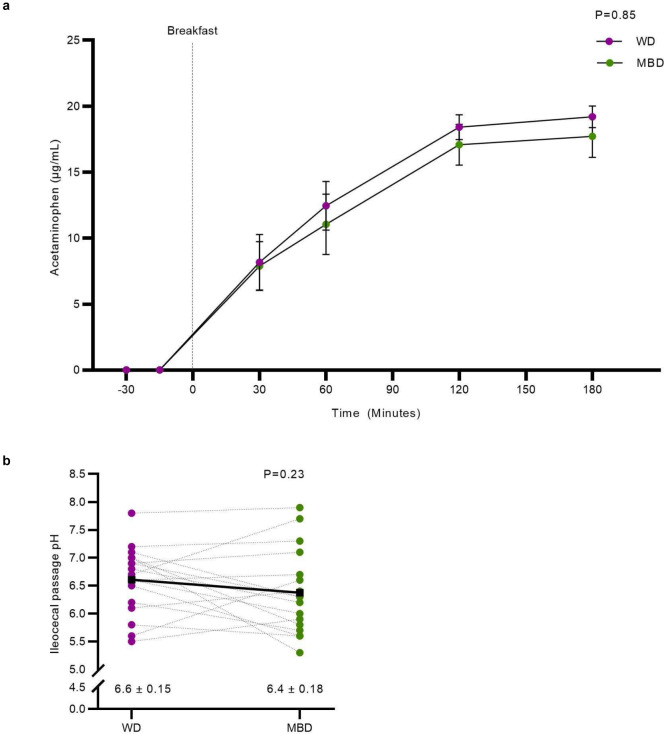

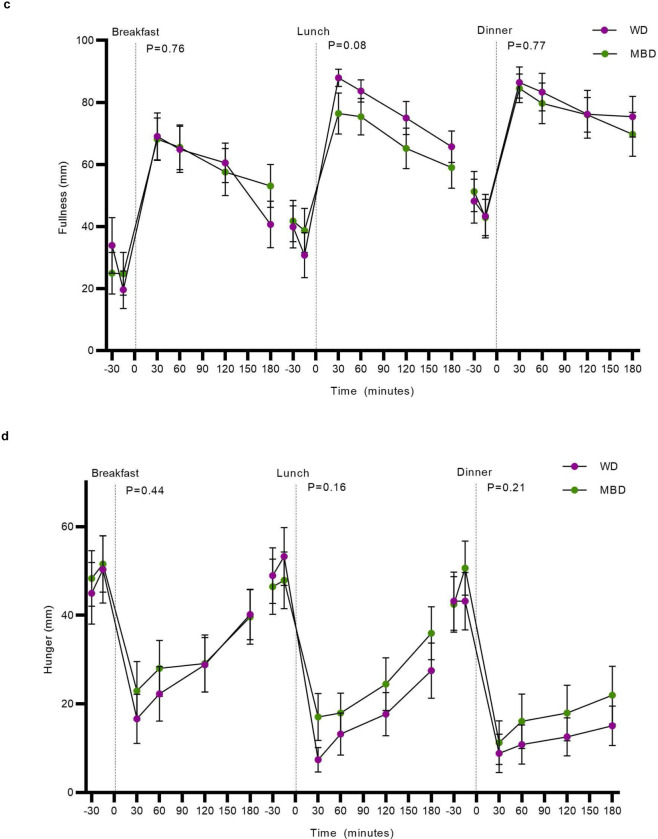

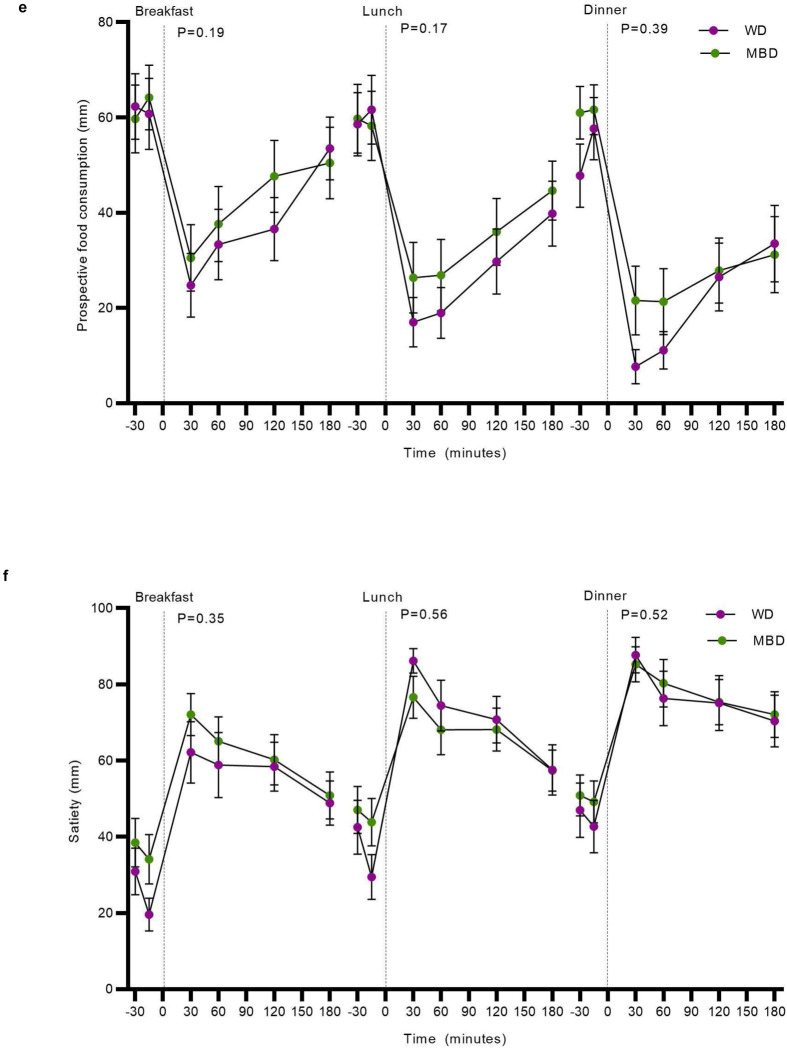

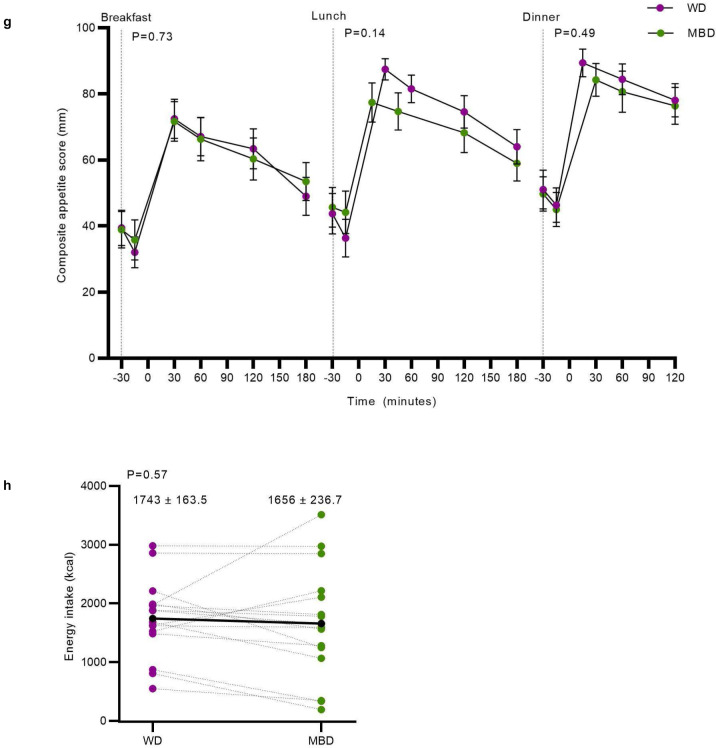
Host response to dietary intervention. **a,** Gastric emptying as evaluated by acetaminophen appearance after a fixed breakfast. **b,** pH within a 1-hour window of the ileocecal passage. **c-g,** Visual analog scale data for subjective ratings of fullness, hunger, prospective food consumption, satiety and a composite appetite score. **h,** Ad libitum energy intake evaluated during lunch and dinner after a fixed breakfast. All data reported as mean ± s.e.m. N=17 per diet for panels a and b; n=16 per diet for panels c-h. MBD—Microbiome Enhancer Diet; WD—Western Diet.

**Extended Data Fig. 5:**
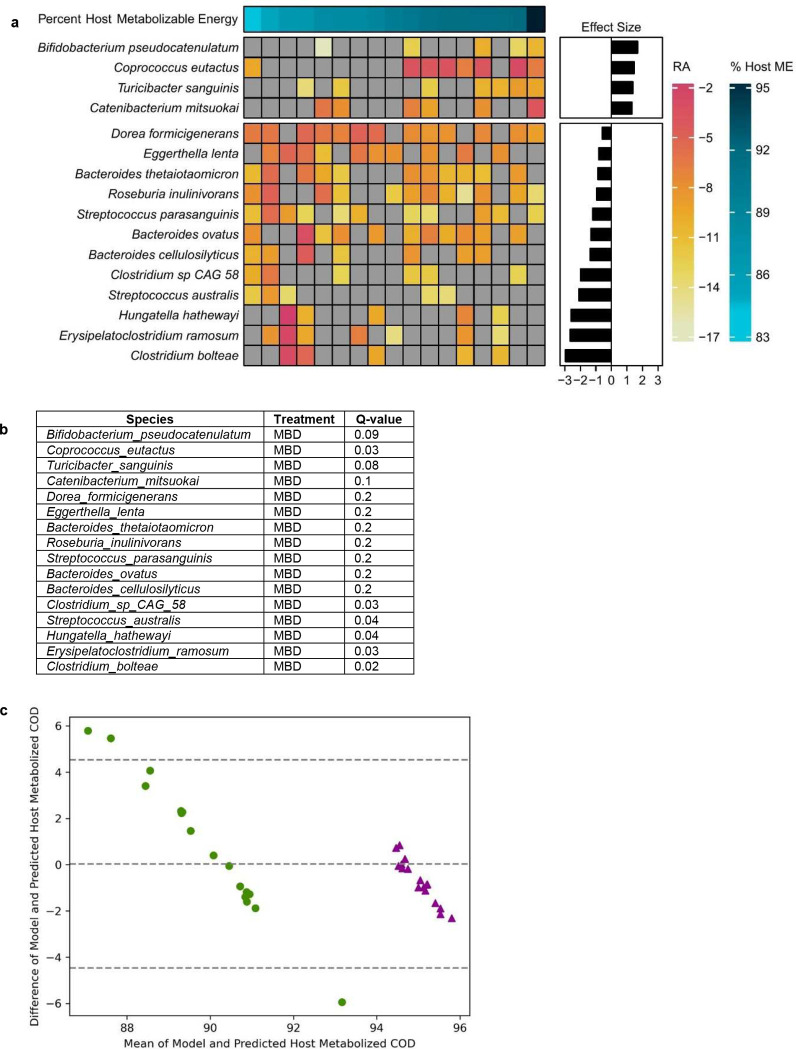

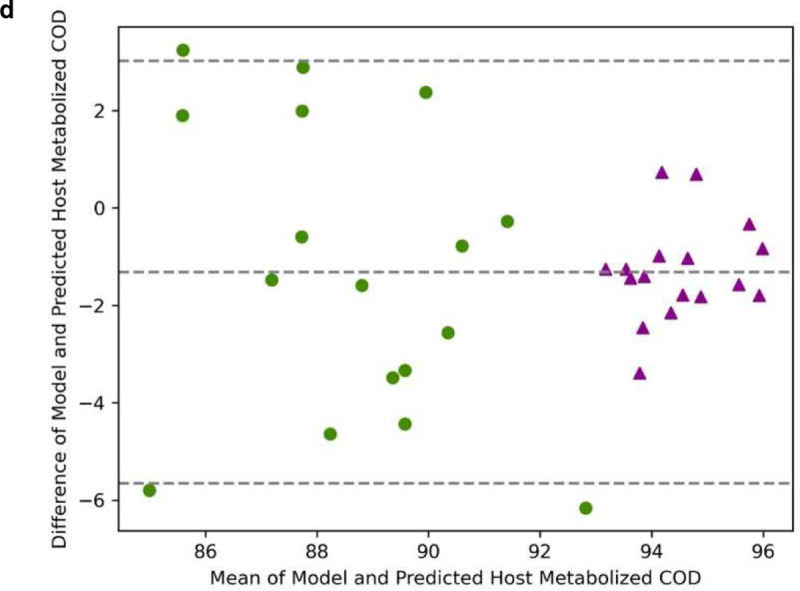
Microbial contributions to host energy balance. **a,** The heatmap shows the associations between host ME and mean RA of each species. Each row is a species and each column is an individual participant. The bar graph shows the effect size of the regression coefficient between the independent variable of host metabolizable energy and each species, from compound Poisson regression models (Q < 0.25) **b,** Q-values for the regression coefficients shown in **a**, for each species (range 0.023 – 0.198). **c,** Bland-Altman plot comparing actual metabolizable energy (absorbed COD) measured for each participant with the model prediction with fixed CTT of 48 h for each participant. **d,** Bland-Altman plot comparing actual metabolizable energy (absorbed COD) measured for each participant with the model prediction with measured CTT for each participant. N=17 per diet for all panels. COD—chemical oxygen demand; ME—metabolizable energy; RA—relative abundance.

**Extended Data Table 1:** Adverse Events

Primary system organ class preferred term [n (%)]	Western Diet	Microbiome Enhancer Diet
	(N=17 participants)	(N=17 participants)
Any class		
Participants: N (%)	6 (35.3%)	5 (29.4%)
Events: N	19	12
Metabolism and nutrition disorders		
Hypoglycemia		
Participants: N (%)	0 (0.0%)	1 (5.9%)
Events: N	0	1
Nervous system disorders		
Headache		
Participants: N (%)	3 (17.7%)	2 (11.8%)
Events: N	3	2
Syncope		
Participants: N (%)	1 (5.9%)	0 (0.0%)
Events: N	1	0
Neuralgia		
Participants: N (%)	1 (5.9%)	0 (0.0%)
Events: N	1	0
Cardiac disorders		
Chest pressure		
Participants: N (%)	1 (5.9%)	0 (0.0%)
Events: N	1	0
Eye disorders		
Dry eye		
Participants: N (%)	1 (5.9%)	0 (0.0%)
Events: N	1	0
Respiratory, Thoracic and Mediastinal Disorders		
Rhinorrhea		
Participants: N (%)	0 (0.0%)	2 (11.8%)
Events: N	0	2
Cough		
Participants: N (%)	1 (5.9%)	1 (5.9%)
Events: N	1	1
Sore throat		
Participants: N (%)	1 (5.9%)	1 (5.9%)
Events: N	1	1
Gastrointestinal disorders		
Constipation		
Participants: N (%)	1 (5.9%)	0 (0.0%)
Events: N	1	0
Nausea		
Participants: N (%)	1 (5.9%)	1 (5.9%)
Events: N	1	1
Vomiting		
Participants: N (%)	1 (5.9%)	0 (0.0%)
Events: N	1	0
Abdominal pain		
Participants: N (%)	2 (11.8%)	1 (5.9%)
Events: N	2	1
Diarrhea		
Participants: N (%)	2 (11.8%)	0 (0.0%)
Events: N	2	0
Gastroesophageal reflux disease		
Participants: N (%)	0 (0.0%)	1 (5.9%)
Events: N	0	1
Skin and subcutaneous tissue disorders		
Rash		
Participants: N (%)	2 (11.8%)	1 (5.9%)
Events: N	2	1
Musculoskeletal and connective tissue disorders		
Pain in extremity		
Participants: N (%)	1 (5.9%)	0 (0.0%)
Events: N	1	0
General disorders and administration site conditions		
Participants: N (%)	0 (0.0%)	1 (5.9%)
Events: N	0	1

**Extended Data Table 2:** Dietary Intake Over 8 Inpatient Days

Nutrient	Target	Western Diet	Microbiome Enhancer Diet
Energy Intake (kcal/8 days)	Equivalence	17,008 ± 683	16,909 ± 700
Carbohydrates (%)	47–52%	48 ± 0.02%	49% ± 0.06 %
Fat (%)	32–37%	35 ± 0.01%	34 ± 0.04%
Protein (%)	15–18%	16 ± 0.009%	17 ± 0.03%
Fiber (g/1000 kcal)	6–10g/1000 kcal vs. 23–30g/1000 kcal	6.4 ± 0.02	26.0 ± 0.06
Resistant Starch, Estimated (g/1000 kcal)	<2g/1000 kcal vs. >8g/1000 kcals	1.2 ± 0	10.3 ± 0

Intake was evaluated by comparing the nutritional composition data from our menu design software to the amount of the provided diet consumed by each participant over the 8 inpatient days when all 3 meals were consumed on our metabolic ward and no modifications to the diets were made for enteroendocrine or food intake testing. As a comprehensive database of resistant starch content of foods does not exist, the contributions for resistant starch were estimated based on published amounts of resistant starch in the foods provided (reference needed). Data reported as mean ± s.e.m.

**Extended Data Table 3:** Baseline Characteristics

Total N	17
Age (years)	30.8 ± 1.9
BMI (kg/m^2^)	25.1 ± 0.52
Female Sex:	8 (47.1)
Race	
Black	11 (64.7)
White	5 (29.4)
Unknown	1 (5.9)
Hispanic/Latino Ethnicity	6 (35.3)
Weight (kg)	70.5 ± 3.0
Waist to Hip Ratio	0.83 ± 0.02
Bristol Stool Scale	3.8 ± 0.10
Bristol Stool Scale	
Type 3	3 (17.65)
Type 4	14 (82.35)
HbA1c (%)	5.0 ± 0.09
TSH (u[IU]/mL)	1.7 ± 0.19
AST (units/L)	25.3 ± 2.4
ALT (units/L)	22.3 ± 3.4
BUN (mg/dL)	11.7 ± 0.64
Creatinine (mg/dL)	0.98 ± 0.06

Continuous variables reported as mean ± s.e.m. Categorical variables reported as N (%).

**Extended Data Table 4:** Differential Relative Abundance by Diet at the Phylum and Family Levels

	P Value	Q Value
**Phylum Level**		
Firmicutes	0.041322	0.7438
Actinobacteria	0.36716	0.921388
Proteobacteria	0.591775	0.921388
Bacteroidetes	0.79006	0.94554
Verrucomicrobia	0.834099	0.94554
Euryarchaeota	0.95604	0.968383
**Family Level**		
Propionibacteriaceae	0.00766	0.402172
Oscillospiraceae	0.004497	0.402172
Actinomycetaceae	0.036966	0.880546
Clostridiales_Family_XIII_Incertae_Sedis	0.092248	0.880546
Acidaminococcaceae	0.051117	0.880546
Bifidobacteriaceae	0.532114	0.969746
Micrococcaceae	0.733506	0.969746
Atopobiaceae	0.817563	0.969746
Coriobacteriaceae	0.725051	0.969746
Eggerthellaceae	0.368079	0.969746
Bacteroidaceae	0.617749	0.969746
Barnesiellaceae	0.679615	0.969746
Odoribacteraceae	0.120954	0.969746
Prevotellaceae	0.274396	0.969746
Rikenellaceae	0.844536	0.969746
Tannerellaceae	0.639694	0.969746
Bacillales_unclassified	0.5078	0.969746
Lactobacillaceae	0.327826	0.969746
Leuconostocaceae	0.622826	0.969746
Streptococcaceae	0.524649	0.969746
Catabacteriaceae	0.278053	0.969746
Christensenellaceae	0.643426	0.969746
Clostridiaceae	0.669668	0.969746
Clostridiales_unclassified	0.639535	0.969746
Eubacteriaceae	0.539811	0.969746
Lachnospiraceae	0.528458	0.969746
Peptostreptococcaceae	0.647885	0.969746
Ruminococcaceae	0.152769	0.969746
Erysipelotrichaceae	0.415012	0.969746
Firmicutes_unclassified	0.838296	0.969746
Veillonellaceae	0.292069	0.969746
Akkermansiaceae	0.83385	0.969746
Desulfovibrionaceae	0.887671	0.972355
Methanobacteriaceae	0.956145	0.984267
Enterobacteriaceae	0.943787	0.984267

## Data Availability

The source data for the study’s primary endpoint can be found in FigShare: https://doi.org/10.6084/m9.figshare.21727889. All other data supporting the findings of this study are available from the corresponding author on reasonable request.
